# A case report of a rare intramuscular granular cell tumor

**DOI:** 10.1186/s13000-015-0390-1

**Published:** 2015-09-17

**Authors:** Natale Porta, Riccardo Mazzitelli, Jessica Cacciotti, Mirko Cirenza, Agata Labate, Maria Grazia Lo Schiavo, Andrea Laghi, Vincenzo Petrozza, Carlo Della Rocca

**Affiliations:** Department of Medico-Surgical Sciences and Biotechnologies, Histology Unit, Sapienza University of Rome, ICOT Hospital, Latina, Italy; Surgical Unit, Casa di Cura “Cappellani Giomi”, Messina, Italy; Pathology Unit, Casa di Cura “Cappellani Giomi”, Messina, Italy; Department of Radiological, Oncological and Pathological Sciences, Radiology Unit, Sapienza University of Rome, ICOT Hospital, Latina, Italy

## Abstract

**Background:**

Granular cell tumors (GCTs) were firstly described by Weber in 1854 and 70 years later by Abrikossoff and classified as benign tumors. Originally considered muscle tumors, they have been identified as neural lesions, due to their close association with nerve and to their immunohystochemical characteristics.

GCTs are uncommon tumors and they may arise in any part of the body; they have been mainly observed in tongue, chest wall and upper extremities; less frequent sites are larynx, gastrointestinal tract, breast, pituitary stalk and the female anogenital region.

Here we report a case of GCT showing an uncommon localization such as the upper third of the right rectus muscle of the abdominal wall.

**Case presentation:**

A 45 year-old woman of Caucasian origin presented to the surgeon with a 6-month history of light pain in the upper third of the abdominal wall.

Radiological exams (Ultrasonography, Computed Tomography and Contrast magnetic resonance imaging) showed a localized in the right rectus abdominis muscle.

After excision, histological and immunohystochemical analysis, with the support of electron microscopy, allowed making diagnosis of granular cell tumor.

**Discussion:**

After fist description by Abrikosoff in 1926 of GCT like mesenchymal tumor of unknown origin, in recent years immunohystochemical techniques definitely demonstrated the histogenetic derivation of GCT from Schwann cells.

Granular cell tumors are rare, small, slow-growing, solitary and painless subcutaneous nodules which behave in a benign fashion, but can have a tendency to recur; in rare cases they can metastasize, when they became malignant; there are some clinical and histological criteria to suspect the malignance of this tumor.

**Conclusion:**

It is important that clinicians, radiologists and pathologists are aware of the clinical presentation and histopathology of GCT for appropriate management, counselling and follow-up. In our case we had a complete radiological, morphological and immunohystochemical characterization of the lesion and a definitive diagnosis of benignity confirmed by electron microscopy.

## Background

Granular cell tumors (GCTs) were firstly described by Weber in 1854 [1] and 70 years later by Abrikossoff [2] and classified as benign tumors. Originally considered muscle tumors, they have been identified as neural lesions, due to their close association with nerve and to their immunohystochemical characteristics.

The highest incidence of GCTs is in the fourth to sixth decades of live and a female preponderance has been reported. Clinically, GCTs are asymptomatic slow-growing tumors, normally present as solitary nodules, smaller than 3 cm; only 10–15 % of patients show multiple synchronous lesions; increased familiar incidence is uncommon, however it has been reported [3].

GCTs are uncommon tumors and they may arise in any part of the body; they have been mainly observed in tongue, chest wall and upper extremities [4, 5]; less frequent sites are larynx, gastrointestinal tract, breast, pituitary stalk and the female anogenital region [5].

GCTs occurring in deep soft tissues are extremely rare, especially those of intramuscular origin [3, 6–11]. Due to their rarity, only few studies are present in the literature describing intramuscular GCTs and they mainly address malignant counterpart [12–15].

The differential diagnosis between benign and malignant lesions can be very difficult, because they can display similar histological phenotypes. In order to discriminate malignant from benign forms, six histological criteria have been established [13]: necrosis, spindling, vesicular nuclei with large nucleoli, increased mitotic activity (>2 mitoses/10 high-power fields at × 200 magnification), high nuclear to cytoplasmic ratio, and pleomorphism. Neoplasms that meet three or more of these criteria are classified as histologically malignant; those displaying only focal pleomorphism but fulfill none of the other criteria are classified as benign, whereas those meeting one or two criteria are classified as atypical.

Here we report a case of GCT showing an uncommon localization such as the upper third of the right rectus muscle of the abdominal wall.

## Case presentation

A 45 year-old woman of Caucasian origin presented to the surgeon with a 6-month history of light pain in the upper third of the abdominal wall. In her medical history, the patient had undergone surgery for tonsillectomy, appendectomy and carpal tunnel syndrome and was suffering from arterial hypertension and thyroid disease, treated with ACE inhibitor and levothyroxine sodium.

Ultrasonography (10 MHz probe), performed in May 2013, showed a 20 mm oval formation localized in the right rectus abdominis muscle with inhomogeneous echogenicity and internal vascular signals.

Computed Tomography (CT) scan with contrast, performed in August 2013, showed inhomogeneous solid nodule with maximum transverse diameter of about 16 mm and a longitudinal extension of 30 mm localized in the right rectus abdominis muscle and characterized by moderate contrast enhancement, without clear demarcation on the profile and light anterior extension in the intra-abdominal adipose tissue.

**Fig. 1:**
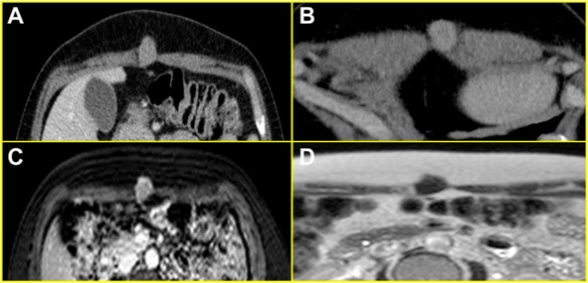
Computed Tomography and Magnetic Resonance Imaging. **a** Axial contrast-enhanced CT image, obtained during the venous phase, showing the nodular lesion, located within the right rectus abdominis muscle. The lesion is homogeneously enhancing, demonstrating a vascularity higher than that of the adjacent muscular tissue; **b** On axial MR T2-weighted the lesion is hypointense compared with adjacent muscle; this finding is indicating a tissue with high cellularity; **c** pre-contrast T1-weighted image and contrast-enhanced fat-suppressed T1 weighted images **d** confirm the vascularity of the lesion

Contrast magnetic resonance imaging, performed in October 2013, displayed a 20 mm oval formation, localized in the right rectus abdominis muscle, hypointense in sequences T1-T2-weighted, homogeneously hyperdense in the late phase with subcutaneous outer profile and intra-abdominal inner profile, without signs of infiltration of the underlying peritoneum (Fig. 1).

Physical examination revealed an oval shaped swelling, motionless on the surface and deep levels, with hard consistency and slightly irregular margins.

At superficial palpation there were no signs of pain; deep palpation evoked moderate soreness at the epigastrium.

In November 2013, the patient underwent to surgical removal of the lesion: surgical access through midline incision of epigastric region showed a not perfectly round hard mass, with adherence zone to the fibers of the right rectus muscle; en block excision was performed with the simultaneous removal of subcutaneous tissue, muscle fascia and parietal peritoneum. No early complication occurred.

**Fig. 2:**
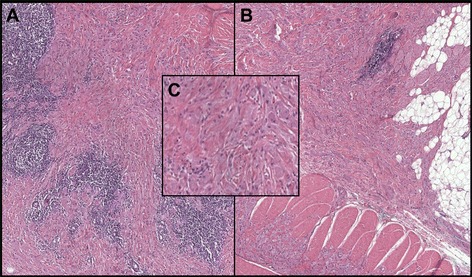
Histological analysis. **a** Medium-power microphotograph showing a proliferation of cellular elements arranged in chains and in nodular aggregates interspersed in dense fibrous stroma and accompanied by multiple nodular foci of lymphoid infiltrate (hematoxylin-eosin, magnification 20×); **b** Medium-power microphotograph of the edges of surgical specimen showing lesion in continuity with striated muscle and adipose tissue (hematoxylin-eosin, magnification 20×); **c** High-power microphotograph showing neoplastic cell with large granular, slightly eosinophilic cytoplasm and small eccentric nuclei (hematoxylin-eosin, magnification 40×)

Macroscopically the surgical specimen was composed of yellow-brown tissue. The cut surface revealed a nodular yellow-grey lesion, with irregular borders and 3 cm of diameter. Microscopically, the histological examination of entire surgical specimen showed a proliferation of cellular elements arranged in chains and in nodular aggregates, characterized by large granular, slightly eosinophilic cytoplasm and small eccentric nuclei. These cellular elements appeared interspersed in dense fibrous stroma and accompanied by multiple nodular foci of lymphoid infiltrate. Marginally, the lesion was in continuity with striated muscle and adipose tissue, so it resulted completely excised (Fig. 2).

**Fig. 3:**
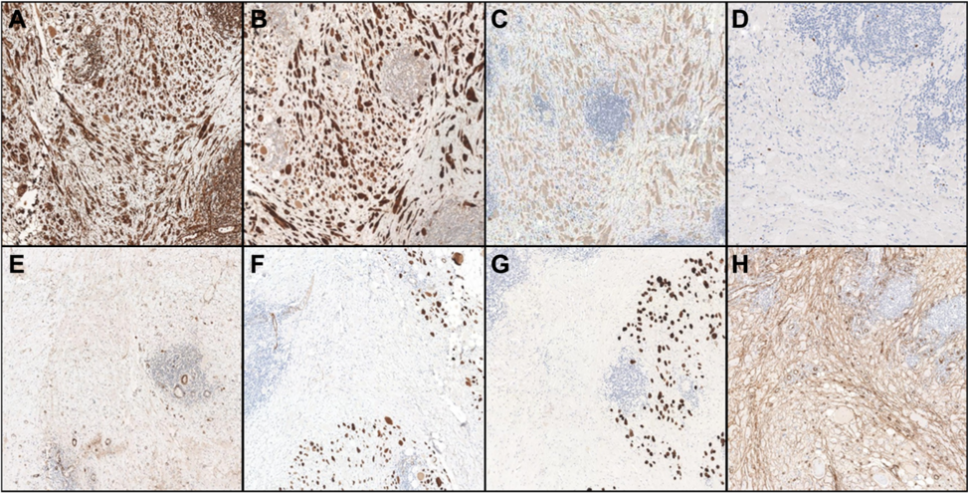
Immunohystochemical analysis. High-power photomicrograph showing immuoreactivity of cellular elements; they resulted positive for Vimentin (**a**), S-100 protein (**b**) and CD68 (**c**), negative for alpha-Smooth Muscle Actin (**e**), Muscle Specific Actin (**f**), Desmin (**g**) and CD34 (**h**) (magnification 20×); the proliferative index (Ki-67) (**d**) was less than 1 % (magnification 40×)

Immunohystochemical analysis of the above mentioned cellular elements showed positivity for Vimentin (clone V9 Novocastra Leica Biosystems), S-100 protein (clone S1/61/69 Novocastra Leica Biosystems) and CD68 (clone 514H12 Novocastra Leica Biosystems), negativity for alpha-Smooth Muscle Actin (clone asm-1 Novocastra Leica Biosystems), Muscle Specific Actin (clone HHF35 Novocastra Leica Biosystems), Desmin (clone DE-R-11 Novocastra Leica Biosystems) and CD34 (clone QBEND/10 Novocastra Leica Biosystems); the proliferative index (Ki-67, clone MM1 Novocastra Leica Biosystems) was less than 1 % (Fig. 3).

**Fig. 4:**
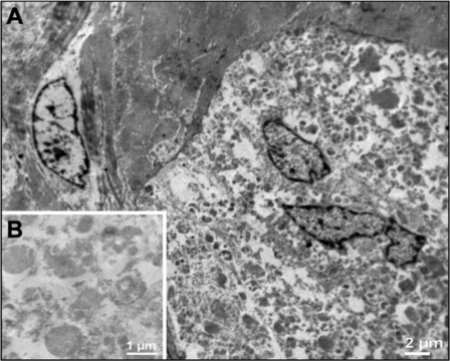
Electron microscopy analysis. **a** Skeletal muscle tissue surrounded by neoplastic cells that show a high number of intracytoplasmic granules of various sizes containing glycogen (magnification 3150×); **b** Detail of intracytoplasmic granules (magnification 10000×)

Electron microscopy showed skeletal muscle tissue surrounded by neoplastic cells that show a high number of intracytoplasmic granules of various sizes containing glycogen (Fig. 4).

According to these morphological and immunophenotypic characteristics, we made the diagnosis of intramuscular granular cell tumor.

## Discussion

In 1926 Abrikossoff et al. reported the first description of the morphological features shared by a spectrum of mesenchymal soft tissue tumours, identifying them as “Granular Cell Tumors” [2].

Following reports focused on their histogenesis, originally believed of skeletal derivation [16] but later proved to be of neural origin, due to the advent of immunohystochemical techniques and electron microscopy.

The positive expression of markers such as S-100, the neuron-specific enolase (NSE) and the histiocytic marker CD68, definitely demonstrated the histogenetic derivation of GCT from Schwann cells [16].

Most of our knowledge regarding GCTs derives from case reports or small case series. Since then, many authors reviewed the reported cases and correlated them with clinical presentations. To date, several controversies about prognostic criteria, treatment strategies and follow-up approaches still exist.

Granular cell tumors are rare and account for approximately 0.5 % of all soft tissue tumors. Females are twice as likely to develop GCT, with African-Americans more often affected than Whites [17]. They occur in both children [18] and adults, with a higher incidence in the fifth decade of life [17]. They generally present as small, slow-growing, solitary and painless subcutaneous nodules which behave in a benign fashion, but can have a tendency to recur [19, 20].

In rare cases they can metastasize, particularly when they arise in deep to fascia or are over 4 cm in diameter [13]. They can be multifocal at presentation, can arise anywhere in the body, typically in the superficial tissues (dermis and sub cutis), along mucosal surfaces and occasionally within skeletal muscle, but they are rarely located in the abdominal wall.

The best radiological modality for the characterization of GCTs is Magnetic Resonance Imaging (MRI) [20]. Benign granular cell tumors are classically iso-intense or brighter than muscle on T1-weighted sequences, round or oval in shape, superficial in location, and 4 cm or less in size. On T2-weighted sequences, the signal from the central portion of the lesion is classically iso-intense to muscle or suppressed fat, with peripheral signal enhancement. Malignant granular cell tumors may demonstrate invasion of adjacent structures and signal intensity characteristics that are often seen in other aggressive neoplasms [19].

We report the case of a patient presenting with a slow-growing, firm nodule in the abdominal wall. After ruling out metastatic origin of the mass by CT scan and MRI, the lesion was surgically removed through an en-block excision with simultaneous removal of subcutaneous tissue, muscle fascia and parietal peritoneum.

Pathologic findings were congruent with a benign granular cell tumour of the abdominal wall.

Although a GCT usually appears as a solitary small nodular growth and follows a benign course, a malignant potential has been described in a few cases, particularly for large lesions, >4 cm in diameter. Comparisons between malignant transformation and de novo primary malignant tumour are based on primary histologic findings and on the clinical course of the patient, especially in recurrent cases during the follow-up period [20]. Malignant GCTs are usually difficult to diagnose because of its rarity [21].

For descriptive purposes these tumours can be classified into two categories: histologically and clinically malignant type, and histologically benign but clinically malignant type.

Although metastasis remains the most important criteria for defining malignancy, not all malignant GCTs truly metastasize. Specific histologic features to predict malignant behaviour include spindling of tumour cells, the presence of vesicular nuclei with large nucleoli, increased mitotic rate, a high nuclear to-cytoplasmic ratio, pleomorphism, and necrosis.

Nevertheless, as malignancy can be definitely proven only by clinical findings, especially metastasis, clinical features such as large size, rapid growth, and invasion into adjacent tissue are reported to be more important criteria of malignancy rather than the histologic features of a histologically potentially malignant tumour.

The malignant form of granular cell tumours is highly aggressive, responds poorly to radiation or chemotherapy and may sometimes have fatal outcomes, if lesions are present in organs such as lung or liver [22].

Local surgical excision, if complete, is curative for benign GCT. The resection margins should be adequate to prevent a misdiagnosis. If resection margins are involved, wider local excision may be recommended to decrease the risk of recurrence.

Recurrence is more likely if the edge of a GCT had an infiltrative and ill-defined pattern, as compared to one with nodular and distinct edges, even with negative margins.

However, in a case series of GCTs in the musculoskeletal system by Rose et al., resection margins or depth of tumour had no correlation with the risk of malignancy or recurrence [23].

## Conclusion

Although GCTs are uncommon and mostly benign, they have a tendency for local recurrence. Some of these cases may be multicentric at presentation. In rare cases metastases or malignant transformation can occur. Wide local excision is the treatment of choice. Hence it is important that clinicians, radiologists and pathologists are aware of the clinical presentation and histopathology of this condition for appropriate management, counselling and follow-up.

In our case we had a complete radiological, morphological and immunohystochemical characterization of the lesion and a definitive diagnosis of benignity confirmed by electron microscopy.

## Consent

Written informed consent was obtained from the patient for publication of this case report and any accompanying images. A copy of the written consent is available for review by the Editor of this journal.

## Abbreviations

GCTs: Granular Cell Tumors
CT: Computed Tomography
RM: Magnetic Resonance

## References

[1] Weber CO (1854). Anatomische Untersuchung einer hypertrophischen Zunge nebst Bemerkungen über die Neubildung quergestreifter Muskelfasern. Virchows Arch A Pathol Anat.

[2] Abrikossoff A (1926). Ueber Myome ausgehened von der quergestreiften willkuerlichen Muskulatur. Virchows Arch A Pathol Anat.

[3] Enzinger FM, Weiss S (2008). Granular cell tumor. Soft Tissue Tumors.

[4] Chaves E, Oliviera AM, Arnaud AC (1972). Retrobulbar granular cell myoblastoma. Br J Ophthalmol.

[5] Dolman PJ, Rootman J, Dolman CL (1987). Infiltrating orbital granular cell tumour: a case report and literature review. Br J Ophthalmol.

[6] Gorelkin L, Costantino MJ, Majmudar B (1978). Granular cell tumor of the abdominal wall musculature. South Med J.

[7] Joshi AH, Aqel NM (2003). Educational case report-self assessment. An anterior abdominal wall tumour. Cytopathology.

[8] An JS, Han SH, Hwang SB, Lee JH, Min BW, Um JW, Lee ES, Park HR, Kim YS (2007). Granular cell tumors of the abdominal wall. Yonsei Med J.

[9] Chaudhry A, Griffiths EA, Shah N, Ravi S (2008). Surgical excision of an abdominal wall granular cell tumor with Permacol® mesh reconstruction: a case report. Int Semin Surg Oncol.

[10] Arai E, Nishida Y, Tsukushi S, Sugiura H, Ishiguro N (2010). Intramuscular granular cell tumor in the lower extremities. Clin Orthop Relat Res.

[11] Panunzi A, D'Orazi V, Toni F, Coppola GA, D'Alessandro V, Pontone S, Pironi D, Ortensi A (2012). Unexpected granular cell tumor in abdominal wall: case report and literature review. Tumori.

[12] Vamsy CM, Smile SR, Ratnakar CR, Veliath AJ (1992). Malignant granular cell tumour. A case report and review of literature. Indian J Cancer.

[13] Fanburg-Smith JC, Meis-Kindblom JM, Fante R, Kindblom LG (1998). Malignant granular cell tumor of soft tissue: diagnostic criteria and clinicopathologic correlation. Am J Surg Pathol.

[14] Chelly I, Bellil K, Mekni A, Bellil S, Belhadjsalah M, Kchir N, Haouet S, Zitouna MM (2005). Malignant granular cell tumor of the abdominal wall. Pathologica.

[15] Tsuchida T, Okada K, Itoi E, Sato T, Sato K (1997). Intramuscular malignant granular cell tumor. Skeletal Radiol.

[16] Apracio SR, Lumsden CE (1969). Light and electron microscopic studies on granular cell myoblastoma of tongue. J Pathol.

[17] Horowitz IR, Copas P, Majmurdar B (1995). Granular cell tumors of the vulva. Am J Obstet Gynecol.

[18] Brooks GG (1985). Granular cell myoblastoma of the vulva in a 6-year-old girl. Am J Obstet Gynecol.

[19] Blacksin MF, White LM, Hameed M, Kandel R, Patterson FR, Benevenia J (2005). Granular cell tumor of the extremity: magnetic resonance imaging characteristics with pathologic correlation. Skeletal Radiol.

[20] Behzatoğlu K, Bahadir B (2007). Malignant granular cell tumor with unusual histological features. Pathol Int.

[21] Mahoney A, Garg A, Wolpowitz D, Mahalingam M (2010). Atypical granular cell tumor-apropos of a case with indeterminate malignant potential. Am J Dermatopathol.

[22] Schmidt O, Fleckenstein GH, Gunawan B, Fuzesi L, Emons G (2003). Recurrence and rapid metastasis formation of a granular cell tumor of the vulva. Eur J Obstet Gynecol Reprod Biol.

[23] Rose B, Tamvakopoulos GS, Yeung E, Pollock R, Skinner J, Briggs T, Cannon S (2009). Granular cell tumours: a rare entity in the musculoskeletal system. Sarcoma.

